# Exercise and Physical Activity in the Therapy of Substance Use Disorders

**DOI:** 10.1100/2012/901741

**Published:** 2012-05-03

**Authors:** Elisabeth Zschucke, Andreas Heinz, Andreas Ströhle

**Affiliations:** Klinik für Psychiatrie und Psychotherapie, Charité-Universitätsmedizin Berlin, Charité Campus Mitte, Charitéplatz 1, 101117 Berlin, Germany

## Abstract

Exercise and physical activity are constantly gaining attention as adjuvant treatment for substance use disorders, supplementing classical pharmacological and psychotherapeutic approaches. The present work reviews studies addressing the therapeutic effects of exercise in alcohol abuse/dependence, nicotine abuse/dependence, and illicit drug abuse/dependence. In the field of smoking cessation, evidence is strong for exercise as an effective adjuvant treatment, whereas no generalizable and methodologically strong studies have been published for alcohol and drug treatment so far, allowing only preliminary conclusions about the effectiveness of exercise in these disorders. A couple of potential mechanisms are discussed, by which exercise may act as an effective treatment, as well as future directions for studies investigating exercise as a treatment strategy for substance use disorders.

## 1. Introduction

Substance use disorders (SUDs), both abuse and dependence, are a common mental health problem with 12-month prevalences ranging from 3.8 to 5.6% [1], causing immense social and economic costs due to physical, psychological, and social comorbidities and consequences (rank 5 of disability-adjusted life years (DALYs) in the European Union, see [1]).

Despite increasingly effective psychopharmacologic and psychotherapeutic intervention strategies, relapse rates are commonly high, resulting in a need for adjuvant therapies that help maintaining abstinence and target physical conditions related to the SUD.

### 1.1. Exercise as Preventive and Therapeutic Intervention

In several cross-sectional studies, levels of exercise (EX) and physical activity (PA) were found to be negatively associated with different mental disorders (e.g., [2]), and higher levels of PA were longitudinally associated with lower onsets of mental disorders [3]. SUDs (alcohol dependence, nicotine dependence, and any SUD) were shown to be less prevalent in physically active subjects [3], and one longitudinal study reported a preventive action of regular PA with regard to alcohol intoxications, alcohol-related problems, and drug use [4].

Additionally, many studies have demonstrated therapeutic effects of EX interventions in other mental disorders, especially depression and anxiety disorders [5–8]. PA and EX may also help to reduce chronic physical conditions which are frequent in patients with mental disorders, especially SUD [9, 10].

This paper aims at subsuming empirical evidence for therapeutic effects of PA and EX in SUD and arriving at conclusions concerning further research and clinical practice.

## 2. Methods

The databases PubMed, Medline, and Web of Science were searched for studies in English or German published between 1970 and 2011 which had investigated any form of EX as therapeutic intervention strategy. Search terms included “exercise,” “physical activity,” “substance use disorder,” “dependence,” “abuse,” “illicit drugs,” “alcohol,” “nicotine,” “cannabis,” “opiate,” “stimulant,” and “cocaine,” in the respective languages.

The bibliographies of all retrieved articles were searched for additional references.

Studies exclusively focusing on exercise as a prevention strategy were excluded.

For nicotine abuse and dependence, only randomized-controlled trials (RCTs) were included into this paper. Since the literature was very limited concerning RCTs on alcohol abuse/dependence and illicit drug abuse/dependence, studies with inadequate control strategies and small samples were also included into this paper.

## 3. Results

In the following sections, studies will be reviewed separately for different SUDs, due to the heterogeneity concerning study designs, methods, and results.

### 3.1. Nicotine Abuse and Dependence

Smoking is very prevalent both in the general population and as a comorbid substance use disorder [45]. Unassisted smoking cessation attempts are mostly unsuccessful, with success rates ranging from 3 to 5% in the 6–12-month followup [46]. Success rates can be increased by behavioral therapy, nicotine replacement therapy (NRT), and medication (e.g., bupropion and varenicline) [47]. 

Since a large number of studies investigated the effects of exercise during and after smoking cessation, only RCTs are listed in Table 1. 

Seventeen RCTs were identified. Fourteen of these trials [11, 12, 14–16, 18–26] studied otherwise healthy subjects, one included patients after acute myocardial infarction [13], another one studied depressed patients [27], and one trial investigated smoking cessation in abstinent alcohol-dependent subjects [17]. Purely female samples were studied in 11 trials [12, 14, 16, 18, 19, 21–24, 26, 27], purely male samples in one trial [13], and mixed samples in five trials [11, 15, 17, 20, 25]. The durations of EX interventions ranged from 5 to 26 weeks, and four studies used EX counseling instead of EX interventions [17, 20, 25, 27]. 

EX interventions were either compared to a standard intervention without EX component [11, 13, 14, 23], to CBT [22], to medication and/or NRT [15, 17, 25], or to a contact control intervention [12, 16, 18–21, 24, 26, 27], and all studies reported smoking-related outcomes such as cigarette craving, withdrawal symptoms, and abstinence and relapse rates, respectively. Compared to a standard intervention, EX was found to improve one or multiple smoking-related outcomes in all four studies. When compared to CBT, EX was found to be as effective concerning abstinence rates (and especially effective when combined with NRT). Two studies reported similar effects for EX and medication/NRT [15, 25], whereas one study [17] found EX augmentation of CBT to be superior to NRT augmentation at posttreatment and similarly effective at 1-year followup. Comparing EX to a control intervention, three studies did not find positive effects of EX on smoking-related outcomes [12, 21, 27], three studies reported a trend towards positive EX effects [16, 24, 26], and two found positive EX effects [18, 20]. 

Evidence is also mixed for secondary outcomes like depression, tension, stress, anxiety, and so forth. on the one hand, and weight gain on the other hand. Concerning emotional changes, two studies reported positive acute or long-term changes [19, 20], two studies did not find EX-induced improvements [26, 27], and one study found even higher tension and anxiety in the EX group at one follow-up time point [12]. Smoking cessation-related weight gain was lower in the EX condition of three studies [18, 22, 23] and higher in one study [27], while three studies [20, 21, 26] reported similar weight gain in EX and control conditions [20, 21, 26].

Taken together, evidence is mixed, but some preliminary conclusions can be drawn concerning favorable effects of EX intervention in smoking cessation. First, EX intervention shows the clearest effects when compared to standard treatment, which becomes more unequivocal, when EX is compared to control groups which offer a similar amount of social support, therapeutic contact, and preoccupation with health-related topics. Second, the majority of studies have shown that EX interventions are as effective as other standard interventions for smoking cessation, such as CBT or NRT/medication. The intensity and frequency of training may be a key point: studies using ≥3 training sessions per week (e.g., [14, 16, 18, 21, 22]) were likely to find fitness gains in the EX group, whereas 1-2 times per week seem not to be sufficient to achieve fitness gains [24]. Although objective assessment of fitness changes were not performed in all studies, five studies that reported fitness gains also reported favorable smoking outcomes [11, 13, 14, 16, 18] compared to three which did not [21, 22, 25], one study reported positive smoking outcomes despite identical increases of fitness in all groups [24], and two studies found neither fitness increases nor favorable smoking outcomes [12, 27]. 

Importantly, three studies concluded that EX adherence rather than the admission to an EX intervention per se predicted smoking abstinence [15, 21, 25, 26], suggesting an important role of motivation, individual resources, and self-efficacy. 

One crucial aspect lies in the moment of implementation of the EX program: one study demonstrated that patients may be overstrained and react with negative effect, when smoking cessation and the EX intervention are realized simultaneously [47]. The implementation of EX a couple of weeks prior to the quit date may be advisable for another reason: EX can serve as a skill to acutely reduce withdrawal and craving symptoms.

A couple of studies addressed this issue (see [48, 49] for a review). In most cases, temporarily abstinent smokers were compared after a short bout of EX versus a control condition (e.g., passive waiting or video). Compared to the control conditions, EX was found to

- reduce the desire to smoke (effect sizes 0.53–2.2 during and after EX, and 0.14–0.74 at the latest follow-up time point);
- reduce withdrawal symptoms (stress, anxiety, tension, irritability, restlessness) and negative mood;
- reduce the anticipation of smoking being rewarding and pleasurable;
- increase the latency period until the next cigarette (effect size 0.85–1.20).

The acute EX interventions ranged from 5 min of isotonic muscle contraction to a brisk one-mile walk. Effects generally appeared very quickly (faster than with oral NRT), lasted between 5 and 50 min, and were not solely explained by distraction, as controlled by different control conditions. 

### 3.2. Alcohol

For the postacute alcohol treatment phase following detoxication, several psychotherapeutic interventions have proven effectiveness, for example, brief interventions (including psychoeducation, motivational interviewing and counseling), and cognitive-behavioral interventions (including cue exposure, self-management strategies, and coping skills) [50].

Furthermore, psychopharmacological treatment with acamprosate or naltrexone significantly increases abstinence rates on the long run [51].

However, the rates of relapse and physical and mental comorbidities are rather high, pointing out the need for adjuvant therapies and long-term life-style modifications.

Compared to smoking, evidence is more limited in alcohol abuse/dependence, and RCTs are extremely rare. Nine studies were identified that investigated the effects of EX programs on abstinence, relapse rates, and/or different associated somatic, emotional, and psychological outcomes (see Table 2).

In these studies, treatment duration ranged from four weeks [28, 33] to four months [31], with training frequencies ranging from three to five times a week. EX interventions were mostly aerobic [28–34, 36], but one study [35] used a holistic “Body-Mind” intervention which prohibits conclusions about EX alone.

Six studies reported drinking episodes, craving, or days of abstinence as substance-related outcomes [28, 30, 32, 34–36], and four of these studies found significantly stronger improvements in the EX group [30, 32, 35, 36], whereas two studies did not find any group differences [28, 34]. Secondary psychological outcomes like depression, anxiety, stress, self-concept, locus of control, and sleep quality, which increased at least in one of the EX conditions, were reported in four studies [28, 29, 31, 33]. In contrast, two studies did not find group differences concerning the reduction of depression [34, 35] and anxiety [34].

Significant increases in fitness were reported in eight studies [28–32, 34–36], which were preserved at 5-month followup in one study [34], whereas one other study [33] did not find significant changes in fitness.

Only one study fulfilled criteria for a RCT [34], whereas the other studies had several methodological limitations. Seven studies included control groups [28, 30–35], whereas one study was a one-group pre-post comparison [29], and one study [36] did not employ control conditions at all. Random assignment of study participants to a treatment condition was performed in four studies [28, 31, 32, 34], whereas one study used a time-staggered control group [33], another study compared samples from different centers [30], and one study did not state their assignment strategy [35].

Sample sizes were small in five studies [28, 31–33, 36], and none of the studies performed intention-to-treat analyses to correct for the high number of dropouts. Four studies did not specify the patients' diagnoses [30, 33, 34] or included subjects without a clinical diagnosis of alcohol abuse or dependence [32]. 

In summary, so far, there is only limited evidence for the efficacy of EX interventions in alcohol rehabilitation. Most cited studies must be interpreted cautiously due to methodological limitations. However, it can be stated that three [34] or four [33] weeks of supervised EX may not be sufficient to induce significant additional changes in anxiety, depression, and abstinence rates and that fitness gains are neither necessary nor sufficient to account for the behavioral and emotional changes reported in most studies.

One possible mechanism of action which is often hypothesized regarding the effects of EX is craving reduction. One study [52] investigated the acute effects of EX in detoxified patients, using a crossover design with 10 min of either moderate or light aerobic EX on a bicycle ergometer. During moderate EX, significant reductions of craving were observed. However, this effect did not continue after the end of the intervention, and there was a trend towards higher baseline levels of craving in the moderate EX condition. Therefore, the craving-reducing activity of EX remains subject to further studies.

An additional study gives information about exercise attitudes and behaviors in a sample of day-clinic patients [53]. Generally, 75% of patients were interested in EX programs, and almost half of the patients stated to exercise regularly (preferably walking, weight lifting, and cycling). EX was appreciated for providing tension relief, stress reduction, and a more positive attitude. Barriers named by the patients included high costs, lack of motivation, time, knowledge, confidence and physical disability.

Hence, adequately powered RCTs are necessary to confirm or disprove beneficial effects of EX interventions in alcohol use disorders, and to disentangle potential mechanisms of action. 

### 3.3. Illicit Substance Abuse/Dependence

Besides substitution therapy, established therapies for illicit drug abuse/ dependence include medication for relapse prevention (e.g., naltrexone) as well as different psychotherapeutic approaches (motivational interviewing, CBT, psychodynamic and systemic approaches, psychoeducation, and social therapy).

So far, no studies satisfying RCT-criteria have been published for this specific population. However, eight studies were identified which investigated therapeutic effects of EX in drug-dependent patients (see Table 3). 

In these studies, treatment duration ranges from to two weeks [44] to six months [42], with training frequencies ranging from several times a day [41] to twice a week [40].

Six studies reported substance-related outcomes like craving, percentage of abstinent subjects, continuous days of abstinence [37, 38, 41–44], which improved with treatment in all six studies. Secondary psychological and social outcomes like depression, anxiety, tension, self-concept, locus of control, employment, and dwelling were reported in five studies [37–39, 41, 42], which generally increased at least in one of the EX conditions.

Fitness increases were reported in three studies [37, 42, 43], whereas the other studies did either not report [38, 40, 41, 44] or find [39] significant changes in fitness. 

All studies suffer from severe methodological limitations which constrain possible conclusions. Only two studies included control groups [38, 41], whereas two studies performed post hoc classifications between improvers and nonimprovers [37, 40], and four studies [39, 42–44] did not employ control conditions at all. 

Sample sizes were very small [39, 40, 42–44] or contained unequal group sizes [38] in six studies, and most studies did not perform intention-to-treat analyses to correct for the high number of dropouts. 

The study participants were mostly in the postacute phase after detoxification, except for the study by Li et al. [41]. Three studies did not specify the patients' diagnoses [39, 40] or included subclinical participants [37]. Furthermore, two studies included culture-specific interventions that were no pure EX interventions [38, 41] which hampers generalizability. In three studies [37, 39, 41], group differences concerning specific outcome variables were found already at the beginning of the study, partly explaining group differences at the end of treatment. Finally, self-reported substance use was not chemically validated in three studies [37, 42, 44].

In summary, evidence is very weak concerning the efficacy of EX as an adjunct therapy in the treatment of illicit drug abuse/dependence. The studies published so far are not methodologically sound and generalizable. Therefore, only a few preliminary conclusions can be drawn pointing towards unspecific benefits of EX, given a certain duration and training intensity of the EX intervention. Well-designed studies using adequate sample sizes and control groups are needed to determine if EX programs are effective for treating SUD and, if so, under which conditions.

## 4. Potential Mechanisms

A couple of mechanisms are discussed when arguing for a beneficial role of EX intervention in psychiatric disorders and especially in SUD. However, most of these mechanisms have not directly been investigated in human SUD population. Therefore, conclusions for mechanisms can cautiously be drawn from animal studies and studies in other human populations, but their translation to SUDs remains speculative at present. Two reviews listed some of these potential mechanisms for alcohol use disorders [54, 55].

### 4.1. Neurochemical Alterations by EX

In alcohol-dependent patients, dysfunctions of dopaminergic, glutamatergic, and opioidergic neurotransmission have been linked to craving and relapse [56]. A large number of animal studies reported acute and chronic EX-induced alterations in different transmitter systems, by modifying transmitter release, reuptake, turnover, or receptor density and sensitivity, respectively. In humans, EX-induced downregulation of postsynaptic serotonin 5-HT_2C_ in panic patients [57] and reductions in prefrontal and limbic/paralimbic opioid receptor availability [58] have been reported. Furthermore, endurance training was shown to induce BDNF secretion [59], which may be linked to neuroprotection and plasticity.

### 4.2. Reduction of Acute Craving

Acute craving for the substance of abuse is a prominent factor of relapse [60]. At least in smoking cessation, there is evidence that EX interventions can acutely downregulate craving and withdrawal-related negative mood [19] (see also Section 3.1).

### 4.3. Endogenous Reward

Many SUD patients know positive, relaxed states only in conjunction with substance consumption. EX can induce pleasurable states by changes in neurotransmission (see above), which can be experienced as an internal reward stimulus [61]. 

### 4.4. Mood Regulation

Negative mood, stress, anxiety, and depressions are associated with a higher risk of relapse [62]. A number of reviews (e.g., [63]) concluded that EX can improve mood and well-being and that this effect is persistent up to 3-4 hours after about of EX [64]. It seems that different types of EX (aerobic or anaerobic) have the ability to improve mood, as long as they are not too intense and competitive, which can in turn worsen negative affect [65].

### 4.5. Reduction of Anxious and Depressive Symptoms

Depression is one of the most prevalent disorders in SUD, and depression is a negative predictor for treatment outcome [66].

Numerous studies supported the effectiveness of EX as a long-term intervention for anxiety and depressive disorders [67], showing that both anaerobic and aerobic trainings are in principle eligible, provided a training duration of approximately 9 weeks [63]. However, evidence is mixed with regard to the optimal intensity—some studies found light-to-moderate EX to be the most effective, others reported the largest effects at higher training intensities.

Finally, it remains controversial whether antidepressant and anxiolytic effects are specific for EX or whether unspecific effects such as therapeutic contact, engaging in health behavior, and social appreciation are mechanisms of action [65].

### 4.6. Stress Reactivity

Subjective stress is a factor often reported to be involved in relapse [62]. Several studies demonstrated that EX can act protectively against everyday-stress [68] and that the stress reaction to a psychosocial stressor is reduced in trained compared to untrained healthy subjects [69]. Also, a single bout of moderate EX was resported to buffer the stress reponse in untrained women [70].

### 4.7. Group Activity and Social Support

A social network that is not primarily related to substance consumption is often hypothesized to be a key factor of relapse prevention. Group EX may help to improve communication skills, conflict management, and frustration tolerance [55, 71].

### 4.8. Coping

Substance use can be interpreted as a maladaptive coping strategy to handle stressful, unpleasant, and difficult situations. In this regard, EX has been proposed to broaden the repertoire of possible behaviors and to provide alternative coping strategies for emotion regulation [72].

### 4.9. Maladaptive Cognitions and Self-Efficacy

Self-efficacy is reduced in many SUD patients who repeatedly experience control loss with regard to their consumption behavior. Some authors assume that supervised training can increase body-related self-efficacy by individual dosage and self-paced progression [73]. However, it remains unclear if these changes generalize to other domains and generally increase self-esteem and reduce expectations of failure.

## 5. Conclusion and Future Directions

The above-cited studies, especially in the field of nicotine abuse/dependence, provide some evidence for positive treatment effects that can be achieved using EX interventions.

Nevertheless, it becomes obvious that evidence is very sparse with regard to alcohol and illicit drugs. Given the self-evident presence of EX programs as an integral part of almost every rehabilitation facility, it has to be pointed out that, so far, no methodologically firm studies are present to verify the long-term benefits of EX interventions with regard to craving, abstinence, relapse, and other psychological variables. Although beneficial effects induced by EX are theoretically plausible, clinically admitted, and highly intuitive, well-designed studies need to be conducted to empirically corroborate these assumptions. Future studies should report effect sizes in order to make EX effects comparable to other active treatments, such as pharmacological or psychotherapeutic interventions.

Generally, patients with poor physical condition are often excluded from studies or drop out early. In the future, more emphasis should be put on the question about (a) how patients with poor physical health can be included into EX programs and (b) how physical health changes moderate SUD-related therapy outcomes. 

In the field of medication abuse, amphetamines, and synthetic drugs, no studies have been published so far. 

Some further questions are of particular interest.

- Which mechanisms of action are particularly important in SUD patients?
- Is there a linear (or curvilinear) dose-response relationship of EX and therapy outcome?
- What is the minimal duration and intensity of an efficient EX program?
- Which types of EX are the most effective, and how is the type of effective EX related to the patients' characteristics and cultural background?
- Which roles do factors like indoors versus outdoors, individual versus group EX, supervised versus nonsupervised training play?
- Does an effective EX intervention necessarily require fitness increases?
- How can patients be motivated to adhere and continue EX programs?

## Figures and Tables

**Table 1:** Randomized-controlled trials investigating EX as an intervention in nicotine abuse/dependence.

First author, year	Sample characteristics	Study design, standard therapy	Exercise intervention (EX)	Control condition(s)	Outcome variables and findings Exercise > control condition	Comments
Hill (1985) [11]	- *N* = 36 untrained smokers (f, m) - >10 cig/day - Ethnicity not reported	- Duration: 5 weeks - Group counseling smoking cessation program (10 sessions)	- Duration: 5 weeks - 2 times/week - 30 min of supervised training + instruction to be as physically active as possible, esp., in case of craving - Aerobic EX (type and intensity not reported)	- Standard therapy	- Trend towards lower number of smoked cigarettes and higher percentage of abstinent patients (not significant) -Higher PA (self report) at the end of treatment and after 1, 3, 6 months	- Small *N * - Lack of equal contact time control - Lack of objective measurement of training effects - EX duration too short to improve fitness

Russell et al. (1988) [12]	- *N* = 42 - heavy smokers (f) - 23 ± 7 cig/day -Ethnicity not reported	- Duration: 1 + 9 weeks - behavioral smoking cessation program (4 × 1 h during first week) + 9 weeks of maintenance	- Duration: 9 weeks - 3 times/week (once supervised, twice alone) - 20–30 min of walking/jogging at 70–80% of max HR	- A: one 30-min educational meeting per week - B: one 30-min contact control meeting per week	- Abstinence rates comparable in all groups at end of treatment and after 3, 6, 18 months - No increase in fitness in EX group - Higher tension/anxiety in EX group after 6 months	- Small *N * - Purely female sample - EX compliance only assessed by self-report

Taylor et al. (1988) [13]	- *N* = 160 - inpatients (m), 10–14 days after acute myocardial infarction (*N* = 68 smokers) - Ethnicity not reported	- Treadmill EX testing	- Duration: 3–26 weeks - A: home EX training - B: medically supervised group EX - Type, frequency, duration, and intensity not reported	- Standard therapy	- No group differences concerning abstinence and relapse rates, but lower number of smoked cigarettes after 26 weeks in groups A and B - significant fitness increases in groups A and B	- Lack of clarity concerning type and intensity of EX intervention - Nonuniform training durations - No smoking cessation intervention - Special sample and high dropout rate due to cardiac events

Marcus et al. (1991) [14]	- Pilot study *N* = 20 untrained smokers (f) of >10 cig/day - Ethnicity not reported	- Duration: 4 weeks - 8 sessions of behavioral outpatient smoking cessation treatment	- Duration: 15 weeks - 3 times/week supervised training - 30–45 min aerobic training (walking, rowing, or cycle ergometry) at 70– 85% max HR - Beginning 3 weeks prior to smoking cessation program	- Standard therapy	- Significantly higher abstinence rates after 1, 3, and 12 months - Significant increases in fitness	- Very small *N * - Purely female sample - Lack of equal contact time control

Hill et al. (1993) [15]	- *N* = 82 heavy smokers (f, m) - ≥30 y of smoking, 28 ± 14 cig/day - Ethnicity not reported	- Duration: 12 weeks - 12 sessions of behavioral training	- Duration: 12 weeks - Weeks 1–4 : 3 times/ week - Weeks 5–8 : 2 times/ week - Weeks 9–12 : once a week + instruction for individual training - 45 min of walking at 60–70% of HR-R - A: EX + behavioral training - B: EX alone	- C: standard therapy - D: standard therapy + NRT	- Significantly higher abstinence rates in groups with behavioral training (A, C, D) - Trend towards higher abstinence in regular versus nonregular walkers within group B	- Low compliance concerning EX program - Lack of objective measurement of training effects - Lack of blinding: therapists = investigator in each group

Marcus et al. (1995) [16]	- *N* = 20 untrained smokers (f) 8–40 cig/day - Ethnicity not reported	- Duration: 12 weeks - 12 sessions of behavioral smoking cessation program	- (As in [12]) Duration: 15 weeks - 3 times/ week supervised training - 30–45 min aerobic training (walking, rowing, or cycle ergometry) at 70– 85% max HR - Beginning 3 weeks prior to smoking cessation program	- One 30-min educational meeting per week (12 sessions, same contact time)	- Descriptively increased 7-day abstinence at end of therapy and at 1–3 month followup - Significant fitness gains at end of treatment	- Very small *N * - Purely female sample - No statistical data analysis

Martin et al. (1997) [17]	- *N* = 205 smokers of > 10 cig/day (f, m) with history of alcohol dependence (≥3 months of alcohol abstinence) - Sample > 90% Caucasian	- Duration: 8 weeks - CBT smoking cessation program or standard ALA intervention + “Nicotine Anonymous” meetings	- CBT counseling + EX ACSM-based EX prescriptions given during last week of CBT: engaging in 3 times of 15– 45 min walking per week and using the laboratory EX equipment	- A: CBT counseling + NRT (2–12 mg/day) - B: standard ALA intervention + “Nicotine Anonymous” meetings	- Significantly higher validated abstinence rates posttreatment (not maintained at 6- or 12-month followup) - Relapse rates for alcohol/drugs not significantly different between groups	- Late implementation of EX program - EX training not supervised - Lack of objective measurement of training effects

Marcus et al. (1999) [18]	- *N* = 281 untrained smokers (f) - 23 ± 10 cig/day - Ethnicity not reported	- Duration: 12 weeks - 12 sessions of behavioral smoking cessation program	- Duration: 12 weeks - 3 times/week supervised training - 30–45 min aerobic training (walking, rowing, or cycle ergometry) at 60– 85% HR-R - Beginning 3 weeks prior to smoking cessation program	- One 30-min educational meeting per week (12 sessions, same contact time)	- Higher abstinence rates at all postquit time points (8, 20, 60 weeks) - Lower weight gain at end of treatment (not maintained at followup) - Significant fitness gains	- Purely female sample - Significant group differences in initial body weight (EX > control)

Bock et al. (1999) [19]	- Two subsamples of [18] - *N* = 62 untrained smokers (f) - Sample > 90% White	- As in [18]	- As in [18]	- As in [18]	- Significant positive acute effects of EX on mood, craving, and withdrawal symptoms (comparison prepost EX sessions) - No significant long-term effects of EX on mood	- Purely female sample - Unequal group sizes (44 : 18) - Only comarisons at baseline and 12 weeks, no time course reported - No between-group comparison reported, but paired *t*-tests within groups

Ussher et al. (2003) [20]	- *N* = 299 untrained smokers (f, m) - 22 ± 9 cig/day - Predominantly white sample (88%)	- Duration: 6 weeks - NRT (15 mg/day) + one weekly CBT group session	- EX counseling: prescription of 5–30 min of EX on ≥5 days per week - EX recommended as self-control strategy	- Health education advice (same contact time)	- Significantly higher abstinence rates after 1 and 2 weeks (no difference after 3, 4, 6 weeks) - Reductions in tension, stress, irritability, restlessness 1 week after end of treatment (partly maintained throughout followup) - Significantly higher self-reported PA at 1, 4, 6 weeks postquit No differences concerning weight gain	- No objective measurement of EX adherence or training effects

Marcus et al. (2005) [21]	- *N* = 217 untrained smokers (f) - 21 ± 9 cig/day - Predominantly white sample (82.5%)	- Duration: 8 weeks - CBT smoking cessation program (8 sessions) +NRT as necessary	- Duration: 8 weeks - once per week: supervised training + prescription to individually train 4 times/week - 30–45 min aerobic training at 50–69% max HR - (resulting in 165 min/week of moderate intensity training) - Beginning 1 week prior to quit day	- Health education advice (same contact time)	- No differences in continuous abstinence or 7-day point prevalence of smoking at posttreatment, and 6 or 12 months followup, except for 7-day point prevalence of smoking at 6-month followup - (EX > control), BUT amount of EX = significant predictor for abstinence - Significant fitness gains - No differences concerning weight gain	- Inclusion of light smokers (≥5 Zig/Tag) - Extremely low abstinence rates in whole sample (<1% after 12 months)

Prapavessis et al. (2007) [22]	- *N* = 142 untrained smokers (f) - >10 cig/day - Ethnicity not reported	- Duration: 12 weeks - Comparison of CBT and EX, each with and without NRT	- Duration: 12 weeks - 3 times/week supervised training - 45 min aerobic training (walking, rowing, or cycle ergometry) at 60–75% HR-R - Beginning 6 weeks prior to quit day - A: with NRT - B: without NRT	- C: CBT with NRT - D: CBT without NRT	- No significant differences in abstinence rates after 3 and 12 months - Short-term improvement of abstinence up to 6 weeks by nicotine replacement (both in EX and CBT) - Significant fitness gains after 12 weeks (back to baseline at 12-month followup) - Delayed weight gain in EX conditions at end of treatment	- Relatively low abstinence rates in all groups after 3 and 12 months

Chaney and Sheriff (2008) [23]	- *N* = 101 smokers (f) - (amount of smoking not reported) - Ethnicity not reported	- Duration: 8 weeks - 1 h/week of behavioral counseling and social support+ NRT	- Duration: 8 Wo - ≥3 times/week - 30 min of circuit training (mixed aerobic/anaerobic) in women's gym	- Standard therapy	- Significantly higher abstinence rates at end of treatment - Significantly lower weight gain	- High drop-out rate - No validation of self-reported smoking - No objective measurement of training effects - No follow-up data reported

Kinnunen et al. (2008) [24]	- *N* = 182 untrained smokers (f) - 19 ± 8 cig/day - Predominantly white sample (81.5%)	- Duration: 19 weeks - 8 brief (10 min) weekly CBT counseling sessions + NRT	- Duration: 19 weeks - Week 1–5: twice a week - Week 6–19: once a week - 30 min of supervised aerobic training (treadmill) at 60–80% max HR - Beginning 3 weeks prior to quit day	- A: standard therapy (CBT counseling) - B: standard therapy (CBT counseling) + health education (same contact time)	- Trend towards higher abstinence rates in EX and control group A at end of treatment and 12 month follow-up - Advantage of EX and control group A in preventing early relapse (at 1 week) - Significant fitness increases in all groups	- High drop-out rate (only 55/182 completed treatment - High relapse rates - No selective training effect in EX group

Prochaska et al. (2008) [25]	- *N* = 407 - smokers (f, m) - 19 ± 8 cig/day - Mixed sample (71% Caucasian)	- Identical treatment for 12 weeks: - NRT + bupropion (2 × 150 mg/day) + 5 group-based smoking cessation sessions	- Weeks 14–16: baseline PA measurement (pedometer) - Week 16–52: relapse prevention program including two counseling sessions (at week 16 and 20) to increase steps 10% biweekly towards 10.000 steps/day - A: standard therapy + 40 weeks EX - B: standard therapy + 40 weeks EX + another 40 weeks of bupropion - C: standard therapy + 40 weeks EX + 40 weeks of placebo	- D: standard therapy without further intervention - E: standard therapy + another 40 weeks of bupropion - F: standard therapy + 40 weeks of placebo	- Increase in PA-predicted abstinence in week 24 - Significant increase in PA in groups A-C compared to groups D–F	- Group differences in terms of abstinence not reported - Relapse prevention program was no pure EX intervention (included motivational aspects, social support, mood, and weight regulation) → no adequate control group - PA increases partly due to group differences in baseline PA (D–F > A–C) - Pedometer data available from only 15% of subjects - No intention-to-treat analysis - No objective measurement of fitness gains

Williams et al. (2010) [26]	- Pilot study: *N* = 60 untrained smokers (f) - >5 cig/day - Sample 85% nonhispanic white	- Duration: 8 weeks - Smoking cessation program with brief (15–20 min) counseling sessions + NRT	- Duration: 8 weeks - 3 times/week - 50 min of aerobic training (treadmill) up to 70% of max HR - minimal interaction with groups members and staff	- Wellness videos (3 times/week30 min) - minimal interaction with groups members and staff	- Trend towards higher prolonged abstinence and lower 7-day point prevalence at posttreatment - No significant group differences with regard to withdrawal symptoms, affect, and weight gain - Correlation of abstinence rates and attended EX sessions - Subjects with high self-efficacy more likely to benefit from EX	- Small *N*, but high compliance - Changes in fitness not reported - EX behavior was not maintained after end of treatment

Vickers et al. (2009) [27]	- *N* = 60 untrained smokers (f) with current depression - 21 ± 8 cig/day - Almost purely white sample	- Duration: 10 weeks - Behavioral counseling for smoking cessation (10 min/week) + NRT (21 mg/day)	- Duration: 10 weeks - 30 min of CBT-based EX counseling once per week, aiming at increasing PA to 30 min/day on ≥ 5 days/week and using EX to overcome acute craving	- 30 min of general health counseling once per week (same contact time)	No group differences with regard to abstinence, mood, and depression More PA reported in EX group, but no changes in fitness measures - Significant weight gain only in EX group (!)	- Concurrent treatment with different medication and psychotherapy for depression - No direct group comparisons reported (just within pre-post differences)

ALA: American Lung Association, CBT: cognitive-behavioral therapy, cig: cigarettes, EX: exercise, f: female, h: hour(s), HR: heart rate, HR-R: heart rate reserve, m: male, max HR: maximum heart rate, min: minutes, *N*: sample size, NRT: nicotine replacement therapy, PA: physical activity.

**Table 2:** Studies investigating EX in the therapy of alcohol abuse/dependence.

First author, year	Sample characteristics	Study design, standard therapy	Exercise intervention (EX)	Control condition(s)	Outcome variables and findings Exercise > control condition	Comments
Gary and Guthrie (1972) [28]	- *N* = 20 - alcohol-dependent patients (m) - Ethnicity not reported	- Inpatient alcohol rehab treatment - Group therapy, recreation programs - Duration and type of therapy not reported - Random assignment to EX or Control	- Duration: 4 weeks - 5 times/week - Incremental running program (1 mile/training day)	- Standard care - (Group therapy, recreation programs)	- No effects with regard to drinking episodes notices by staff	- Small *N* - Lack of equal contact time control - Duration and type of therapy not reported - No direct alcohol-related outcomes reported - No follow-up data reported
					(no other alcohol-related outcomes reported) - Significant gains in cardiovascular fitness and self-cathexis scale, Significantly reduced sleep disturbances	

Frankel and Murphy (1974) [29]	- *N* = 214 - alcohol-dependent patients (m) - Ethnicity not reported	- Inpatient alcohol rehab treatment - Group psycho-therapy, physical fitness program, work assignment, education, family counseling, individual therapy - Duration: 12 weeks	- Duration: 12 weeks - 5 times/week, 1 h each - Warm-up, individual strengthening activities, 20 m in of cardiovascular training (group walk or run)	None	- No alcohol-related outcomes reported - significant gains in cardiovascular fitness, reductions in self-reported depression and paranoia	- One-sample pre-post comparison - Lack of control condition to control for general treatment/recovery effects - No alcohol-related outcomes reported - No follow-up data reported

Sinyor et al. (1982) [30]	- *N* = 58 - patients (m, f) (diagnoses not reported) - Ethnicity not reported	- Inpatient alcohol rehab treatment - Daily group therapy led by abstinent alcoholics - Type of therapy not reported Duration: 6 weeks	- Duration: 6 weeks - 5 times/week, 1 h each - Stretching, calisthenics, muscle-strengthening EX, running or cross-country skiing	- Control group with standard care in different therapy center	- At 3-month followup, significantly higher abstinence rates (self- report, validated by family members or colleagues) - Significant fitness gains	- Comparison of patients from different study centers (effects of patient or treatment characteristics interfering with effects of EX) - Lack of randomization

Weber (1984) [31]	- *N* = 46 - alcohol-dependent patients (m) - Ethnicity not reported	- Inpatient alcohol rehab treatment - Type of therapy not reported - Duration: 4 months - Randomized assignment to treatment group	- Duration: 4 months - 3 times/week - Running with increasing intensity and duration (individually adjusted)	- Standard therapy (type not reported)	- No alcohol-related outcomes reported - Significant training effects: almost all patients were able to run ≥ 1 h at the end of treatment - Significantly stronger reductions of stress (self-developed scale), nonsignificant improvements regarding state anxiety (STAI), depression, psychosomatic symptoms, coping, well-being	- Small *N* - Lack of equal contact time control - No alcohol-related outcomes reported - Lack of intention-to-treat analysis to correct for high dropout rate (only 26/46 patients included in analyses) - No follow-up data reported

Murphy et al. (1986) [32]	- *N* = 48 - students (m) classified as “heavy social drinkers” - Ethnicity not reported	- Daily journals recording 15 different variables - Duration: 16 weeks: 2 weeks baseline, 8 weeks intervention, 6 weeks followup - Randomized assignment to condition	- Duration: 8 weeks - 3 times/week (+ instruction to train at least one time/week on their own) - 30 min of running at individual intensity	- Control 1: standard intervention (daily journals) - Control 2:3 times/week supervised meditation	- Significantly stronger reduction in alcohol consumption during treatment phase (also trendwise during followup) - Only alcohol consumption on weekdays affected, not on weekends - Significant fitness gains	- Small *N * - Subjects without clinical diagnosis of alcohol abuse/dependence - Only self-report of drinking behavior - Drinking behavior uncorrelated with fitness gains → general lifestyle modification? - Subjects with high treatment compliance from control 2 improved as much as subjects in EX group

Palmer et al. (1988) [33]	- *N* = 27 - “alcoholic patients” (m, f) (diagnoses not reported) - Predominantly white sample (92%)	- Inpatient alcohol rehab treatment (influenced by AA philosophy) - Duration: 4 weeks	- Duration: 4 weeks - 3 times/week - 20–30 min Walking/running at 60–80% maximum HR	- Standard care without EX (time-staggered)	- No alcohol-related outcomes reported - No fitness gains in EX group - Significantly lower anxiety and depression in EX group at the end of treatment	- Small *N * - Lack of equal contact time control - EX duration too short to improve fitness - No alcohol-related outcomes reported - No follow-up data reported

Donaghy (1997) [34]	- *N* = 165 “alcoholic patients” (m, f) - (diagnoses not reported) - Ethnicity not reported	- Multicenter study: inpatient and outpatient treatment programs of different kinds and durations	- Duration: 3 weeks supervised EX, followed by 12 weeks home-based EX - 3 times/week - 30 min of aerobic and muscle-strengthening training following ACSM guidelines	- Duration: 3 weeks of supervised gentle stretching and breathing exercises, followed by 12 weeks home-based training - 3 times/week, 30 min each	- No significant differences in abstinence rates - Significant higher improvement in power, fitness, body self-perception, and self-esteem after 15 weeks - Power and fitness gains maintained at 5-month followup - No differences in body weight and resting pulse - Anxiety and depression equally reduced in both groups	- Diagnoses and type of therapy not reported - Lack of intention-to-treat analysis to correct for high number of dropouts at followup

Ermalinski et al. (1997) [35]	- *N* = 90 - veterans (m) - Ethnicity not reported	- Inpatient alcohol rehab treatment - daily group psychotherapy, didactic sessions, incentive therapy - Duration: 6 weeks	- Duration: 6 weeks “Body-Mind-Component” - 5 times/week, 1.5 h each - Fitness component including Yoga, incremental jogging in place up to 20 min; other components: motivational aspects, responsibility for health elements	- Standard care without “Body-Mind-Component”	- Significantly reduced craving in “Body-Mind-Component” group - Only partial fitness gains - No impact of “Body-Mind-Component” on depression, body satisfaction - Significant increase of internal locus of control and responsibility for health	- No pure EX program: unclear which part of the “Body-Mind-Component” is related to reported changes - Nongeneralizable sample (veterans)

Brown et al. (2009) [36]	- *N* = 19 - alcohol-dependent patients (m, f) after detoxification (mean 19 days) - Ethnicity not reported	- Pilot study, outpatient alcohol treatment program (no details reported)	- Duration: 12 weeks - Once per week supervised, including CBT-based EX counseling, 2-3 times/week alone - 20–40 min (gradually increasing) of aerobic training (treadmill, ergometer) at 50–69% max HR	None	- Significantly higher rate of abstinent days at end of treatment and 3-month followup - Significantly increased fitness and decreased BMI at end of treatment (no difference at 3-month followup)	- Very small *N * - Lack of control group, therefore effects not explained by EX alone

AA: alcoholics anonymous, ACSM: American College of Sports Medicine, CBT: cognitive-behavioral therapy, EX: exercise, f: female, h: hour(s), HR: heart rate, HR-R: heart rate reserve, m: male, max HR: maximum heart rate, min: minutes, *N*: sample size, PA: physical activity.

**Table 3:** Studies investigating EX in the therapy of illicit drug abuse/dependence.

First author, year	Sample characteristics	Study design, standard therapy	Exercise intervention (EX)	Control condition(s)	Outcome variables and findings Exercise > control condition	Comments
Collingwood et al. (1991) [37]	- *N* = 74 teenagers (mean 16.8 y) - at-risk of drug abuse or abusing drugs or drug-dependent (m, f) - Ethnicity not reported	- *N* = 54 secondary school at-risk classes drug prevention program - *N* = 11 community-based substance abuse counseling agency program duration - *N* = 6 weeks *N* = 9 inpatient chemical dependency unit treatment - Duration: 8 weeks	- Duration: 9 Weeks - 1-2 times/week supervised group EX, 2 individual weekly training sessions - 60–90 min, stretching, strength development, individual aerobic EX	- None - statistical comparison between subjects with (*N* = 38) versus without (*N* = 36) significantly increased fitness (“improvers” versus “non-improvers”)	- “Improvers”: significantly fewer multiple drug users and alcohol uses per week, and higher abstinence rate (but no differences concerning the use of individual substances) - Significantly higher flexibility and strength, and lower body fat Stronger decrease of depression and anxiety, more positive self-concept	- No experimental design - Post hoc classification possibly confounded with other variables (EX adherence and abstinence may depend on the same external factors; causality is not given) - Heterogeneity of diagnoses and institutions - Significant differences between groups concerning initial fitness levels, self-concept, abstinence rates, depression, and anxiety - Drug use only assessed by self-report

Burling et al. (1992) [38]	- *N* = 218 veterans with drug and/or alcohol abuse or dependence (m) - Mixed sample (50% Black, 41% White, 9% other)	- Inpatient rehabilitation program for homeless substance-dependent veterans - Group-based CBT skills training - Duration ≥ 30 days	- Duration ≥ 30 days (optional continuation as outpatient) - Softball team (*N* = 34): 2 trainings, one game (men's city league), one team meeting/week	- Two control cohorts: - A: *N* = 102, patients treated at the same time who chose not to participate in EX - B: *N* = 82, patients treated 1 year prior to EX cohort	- Significantly higher abstinence rate in softball cohort at 3-month followup - Trend for higher employment and dwelling - Significantly higher treatment duration and completion	- Unequal group sizes (32 : 102 : 82) - Self-selection of participants, no randomization - Lack of pure EX intervention includes social interaction, team-building, and time structuring - Lack of equal contact time control group controlling for these factors - Cultural specificity of softball (lack of generalizability)

Palmer et al. (1995) [39]	- *N* = 45 patients with “problems with alcohol, cocaine or other drugs” (m, f) - (diagnoses not specified) - Mixed sample (60% Black, 40% White)	- Inpatient rehabilitation program - Duration: 28–45 days - Random assignment to one of three EX conditions	- Duration: 4 weeks - 3 times/week, 30–40 min supervised EX - A: step-aerobic program at 60% max HR (aerobic training) - B: body-building program (anaerobic strength training) - C: circuit training (mixed aerobic and anaerobic training)	None	- No drug-related outcomes reported - No fitness gains in either group after 4 weeks - Significantly reduced depression scores in group B (bodybuilding), no changes in groups A and C - No changes in blood pressure or resting pulse	- Small *N * - Heterogeneity and lack of clarity concerning the diagnoses - Initial differences between groups concerning depression scores → significant reductions in group B possibly explained by higher initial scores - Training in group B in teams of 2-3, in A and C alone → social confounds - EX duration too short to improve fitness - Lack of intention-to treat analysis to correct for high number of dropouts - No follow-up data reported

Williams (2000) [40]	- *N* = 20 “asymptomatic offenders” (m, f) consuming metamphetamine, alcohol, cannabis, or other drugs - (diagnoses not specified) - Mixed sample (70% Caucasian, 20% Hispanic, 5% Afro-American, 5% native American)	- Outpatient community-based treatment program with 2 weekly sessions for relapse prevention - Duration: 12 weeks	- Duration: 12 weeks - Daily journals recording PA and optional 2 times/week of supervised muscle strengthening training	- None - post hoc comparison between program “completers” and “non-completers”	- No drug-related outcomes reported - “Completers” more involved into EX behavior even before the treatment - Subjects agreed that EX was effective in preventing them from relapse	- Very small *N* (9 dropouts), no meaningful statistic - No group assignment, no control group - Post hoc classification possibly confounded with other variables (EX adherence and abstinence may depend on the same external factors; causality is not given) - Self-selection of participants

Li et al. (2002) [41]	- *N* = 86 heroin-dependent patients (m) - Ethnicity not reported	- Mandatory inpatient treatment (detoxification) - Duration: 1–3 months	- Duration: 10 days - 4-5 times/day - 25–30 min Pan Gu Qigong	- A: 10 days of medication with lofexidine HCl - B: detoxification without special treatment except for PRN medication against severe withdrawal symptoms	- Fewer withdrawal symptoms and earlier negative morphine test in Qigong group - Fewer symptoms of anxiety in Qigong group compared to control groups A and B	- Ethical problems (mandatory treatment) - Lack of specific EX intervention (multifaceted intervention) - Cultural specificity of Pan Gu Qigong (lack of generalizability) - Initial group differences on day 1 concerning withdrawal symptoms → faster reductions possibly explained by lower initial scores - No follow-up data for postacute phase reported

Roessler (2010) [42]	- Pilot study *N* = 38 patients dependent of cannabis, opiate, medication, heroine, cocaine, and/or amphetamine (m, f) - Ethnicity not reported	- Day clinic for substance dependent patients, - (type of treatment not reported) - Duration: 2 and 6 months, respectively	- Duration: 2 and 6 months, respectively - 3 times/week 2h of endurance training, strengthening EX, and team sports (badminton, volleyball)	None	- Improvements in subjective control, craving, role of the substance - Significantly improved fitness at end of treatment - subjective reports: increased fitness reduces withdrawal symptoms and improves body perception, vigor, sleep quality, and self-confidence	- Very small *N*, very high dropout rates (*N* = 17 for pre-post comparison) - Sample selected by clinic staff based on physical condition, compliance, and so forth. - Lack of control group → changes cannot be attributed to EX - No validation of self-reported substance use

Brown et al. (2010) [43]	- Pilot study *N* = 16 untrained patients dependent of alcohol, cannabis, cocaine, opiates, and/or sedatives (m, f) - Mixed sample (81% Caucasian, 13% Afro-American, 6% Hispanic)	- Different concurrent treatments for substance use (inpatient, outpatient, day-clinic, individual sessions with psychiatrists) - Duration: not standardized	- Duration: 12 weeks - 1 supervised and 2-3 individual training sessions per week, progressively 20–40 min of moderate aerobic training (55–69% max HR) - 1 brief weekly CBT intervention to increase motivation for PA - incentive component for EX adherence	None	- At end of treatment, 66% of patients abstinent, with significantly lower relapse rates in patients who had attended at least 75% of EX sessions - Significantly increased fitness after 12 weeks	- Very small *N*, high dropout rate - Lack of control group → changes cannot be attributed to EX alone - EX adherence and abstinence may depend on the same external factors (causality not given)

Buchowski et al. (2011) [44]	- Pilot study *N* = 12 untrained cannabis-dependent patients not seeking treatment for their cannabis dependence - Ethnicity not reported	None	- Duration: 2 weeks - 5 times/week supervised training session (total of 10) - 30 min moderate treadmill walking/ running	None	- Cannabis consumption significantly reduced compared to baseline during intervention and at 2-week followup - Significantly reduced craving after each training session	- Very small *N * - Lack of control group, no substance-related intervention - EX duration too short to improve fitness - Only self-report of cannabis consumption - Motivation of patients unclear: ostensibly no desire for change concerning cannabis consumption, but very high adherence rates

AA: alcoholics anonymous, ACSM: American College of Sports Medicine, CBT: cognitive-behavioral therapy, EX: exercise, f: female, h: hour(s), HR: heart rate, HR-R: heart rate reserve, m: male, max HR: maximum heart rate, min: minutes, *N*: sample size, PA: physical activity.
